# The synthesis and structural properties of a chlorido­bis­{*N*-[(4-meth­oxy­phen­yl)imino]­pyrrolidine-1-carboxamide}­zinc(II) (aceto­nitrile)­trichlorido­zincate coordination complex

**DOI:** 10.1107/S2056989023010447

**Published:** 2024-01-01

**Authors:** Laxmi Tiwari, Kristopher V Waynant

**Affiliations:** a Department of Chemistry, University of Idaho, 875 Perimeter Dr. MS 2343, Moscow, ID 83844, USA; University of Massachusetts Dartmouth, USA

**Keywords:** crystal structure, coordination complex, zinc(II), aryl­azoformamide

## Abstract

The title complex, [ZnCl(C_12_H_15_N_3_O_2_)_2_][ZnCl_3_(CH_3_CN)], was synthesized and its structure was fully characterized through single-crystal X-ray diffraction analysis. The complex crystallizes in the ortho­rhom­bic system, space group *Pbca* (61), with a central zinc atom coordinating one chlorine atom and two pyrrolidinyl-4-meth­oxy­phenyl azoformamide ligands in a bidentate manner, utilizing both the nitro­gen and oxygen atoms in a 1,3-heterodiene (N=N—C=O) motif for coordinative bonding, yielding an overall positively (+1) charged complex. The complex is accompanied by a [(CH_3_CN)ZnCl_3_]^−^ counter-ion. The crystal data show that the harder oxygen atoms in the heterodiene zinc chelate form bonding inter­actions with distances of 2.002 (3) and 2.012 (3) Å, while nitro­gen atoms are coordinated by the central zinc cation with bond lengths of 2.207 (3) and 2.211 (3) Å. To gain further insight into the inter­molecular inter­actions within the crystal, Hirshfeld surface analysis was performed, along with the calculation of two-dimensional fingerprint plots. This analysis revealed that H⋯H (39.9%), Cl⋯H/H⋯Cl (28.2%) and C⋯H/H⋯C (7.2%) inter­actions are dominant. This unique crystal structure sheds light on arrangement and bonding inter­actions with azo­formamide ligands, and their unique qualities over similar semicarbazone and azo­thio­formamide structures.

## Abstract 2

The title compound was formed from the reaction of zinc(II) chloride and pyrrolidine-4-meth­oxy­phenyl azoformamide ligands. The ligands associated in a bidentate manner and the recrystallized complex indicated that the central zinc(II) gave a chloride to a second zinc(II) chloride compound and associated with an aceto­nitrile solvent to give a aceto­nitrile­tri­chlorido­zincate counter-ion.

## 1. Chemical context

Herein is presented pyrrolidinyl-4-meth­oxy­phenyl­azo­form­amide, an aryl­azoformamide (AAF), acting as a ligand through its 1,3-heterodiene N=N—C=O motif to form a coordination complex with a zinc(II) metal atom. AAFs belong to the semicarbazone ligand family and there have been numerous reports and reviews of their use as ligands (Casas *et al.*, 2000 ▸; Mir *et al.*, 2024 ▸; Padhyé & Kauffman, 1985 ▸). Semicarbazones and thio­semicarbazones have the capability to coordinate with late transition metals (*e.g.* Cu, Pd, Zn, and Ni) and these complexes have found applications due to their thermal stability, noteworthy biological properties, and high synthetic flexibility (Casas *et al.*, 2000 ▸; Garg & Jain, 1988 ▸; Kasuga *et al.*, 2003 ▸; Siji *et al.*, 2010 ▸). Extending from the semicarbazones, numerous zinc(II) complexes have been reported to form with Schiff base ligands, exhibiting applications in catalysis and demonstrating anti­bacterial and anti­cancer properties (Kasuga *et al.*, 2003 ▸; Pieczonka *et al.*, 2014 ▸). AAFs, however, have been underexplored as ligands yet have been indicated as reagents for the Mitsunobu reaction (Hirose, *et al.*, 2018 ▸). As ligands, AAFs differ from semicarbazones in the manner of the 1,3-heterodiene motif; where the semicarbazones form a five-membered coordination ring through a Schiff base of type *R*—C=**N**—NH(*R*)—C=**O**, the azoformamide uses the *R*—**N**=N—C=**O** to generate the five-membered metal-chelate. For the coordination process described herein, two AAF ligands are coordinated by zinc(II) chloride, displacing a chloride that is then taken on by a separate aceto­nitrile-coordinated zinc(II) chloride, creating an aceto­nitrile zinc(II) trichloride anion and resulting in a 2:1 ratio of ligands to the metal atom in the formed cationic complex. The ligands remain neutral while the resultant zinc inter­action is similar to the complexes formed with azo­thio­formamide ligands when bound to copper(I) and silver(I) coordination complexes (Groner *et al.*, 2019 ▸; Johnson *et al.*, 2017 ▸; Pradhan *et al.*, 2023 ▸). 
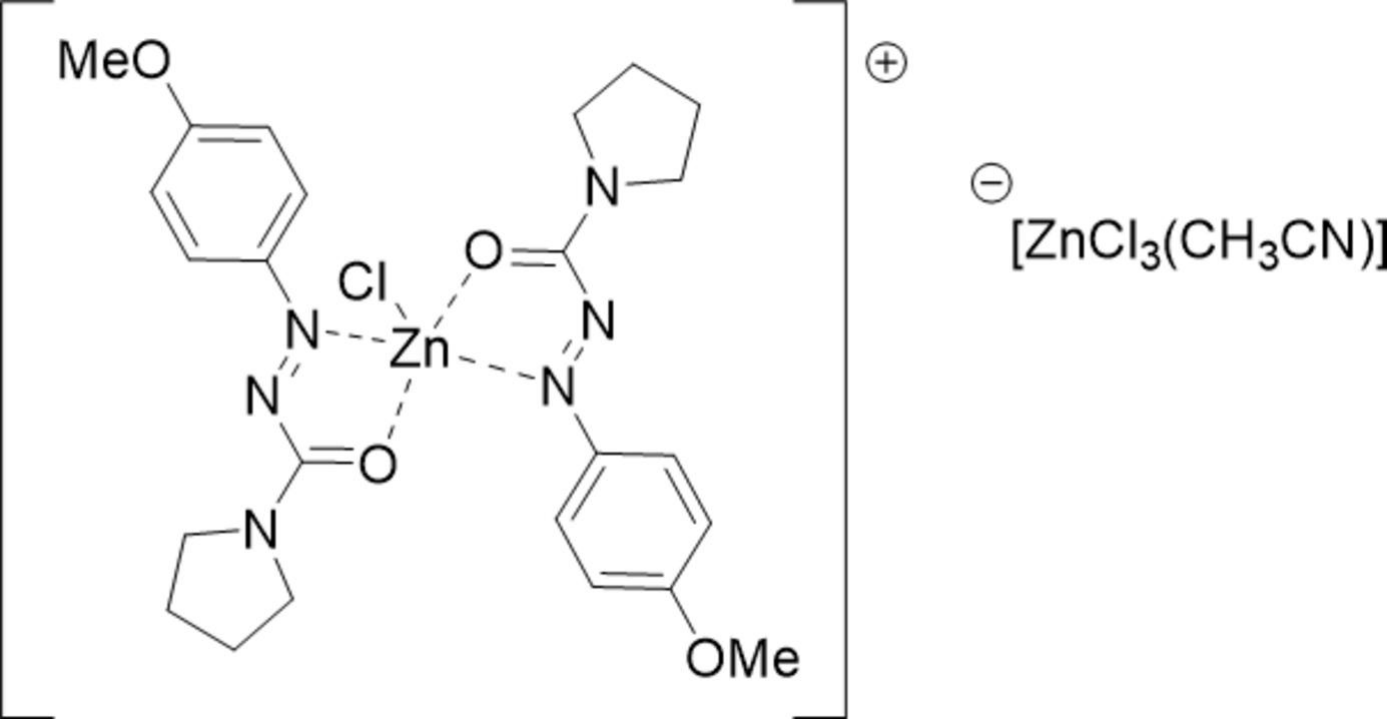


## 2. Structural commentary

**Figure 1:**
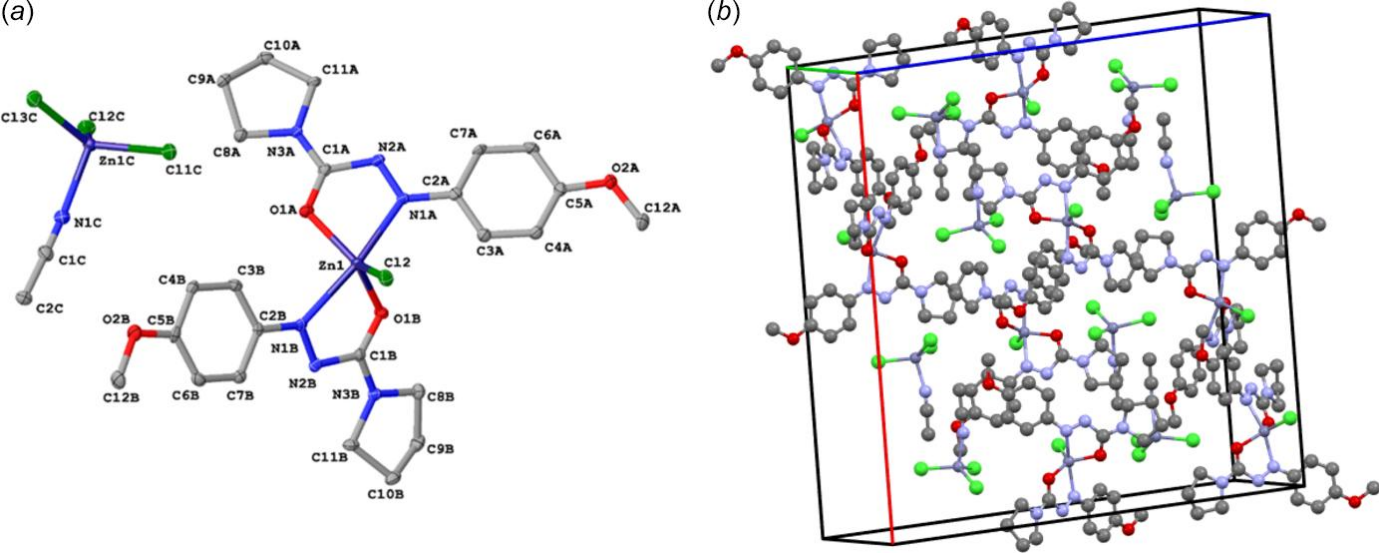
(*a*) A view of the mol­ecular structure of the title complex in its found asymmetric unit, with atom labeling. All displacement ellipsoids are drawn at the 50% probability level. (*b*) Crystal packing diagram of the title complex in ball-and-stick format.

The X-ray crystal structure of the asymmetric unit of the title complex **1** and its packing structure are shown in Fig. 1 ▸. This complex crystallizes in the ortho­rhom­bic space group *Pbca* (61). In this structure, the Zn^II^ ion coordinates two nitro­gen atoms and two oxygen atoms of two pyrrolidine *p*-meth­oxy phenyl­azoformamide mol­ecules along with one chlorine atom, providing a distorted trigonal–bipyramidal shape and rendering the complex positively charged. This positive charge is counterbalanced by the presence of [(CH_3_CN)ZnCl_3_] as counter-ion. Notably, the bond length of Zn1 and the attached chlorine atom (Cl2) is 2.2202 (10) Å; Zn1 and the O1 atom of the azoformamide are measured at 2.002 (3) and 2.012 (3) in the two ligands whereas the Zn1—N1 bonds are 2.207 (3) and 2.211 (3) Å.

## 3. Supra­molecular features, Hirshfeld surface analysis and 2D fingerprint plots

In the crystal, the positive complexes alternate with inverted [(CH_3_CN)ZnCl_3_] counter-ions, as seen in Fig. 1 ▸ *b*.

**Figure 2:**
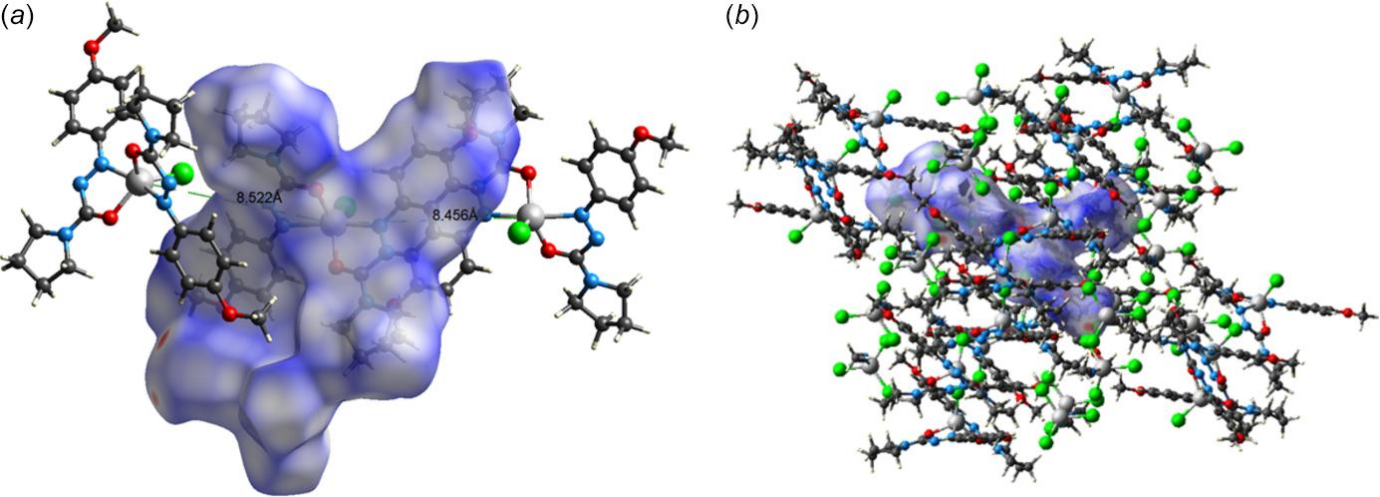
(*a*) Hirshfeld surface of the title compound mapped over *d*_norm_ with Zn—Zn bond lengths in Å. (*b*) Arrangement of the compound in the crystal with inter­actions indicated.

In order to visualize the inter­molecular inter­actions, a Hirshfeld surface (HS) analysis was carried out using *Crystal Explorer 17.5* (Spackman *et al.*, 2021 ▸), which was also used to generate the associated two-dimensional fingerprint plots. Red and blue dots on the Hirshfeld surfaces (Fig. 2 ▸) indicate intermolecular contacts with distances shorter and longer than the van der Waals radii, respectively.

**Figure 3:**
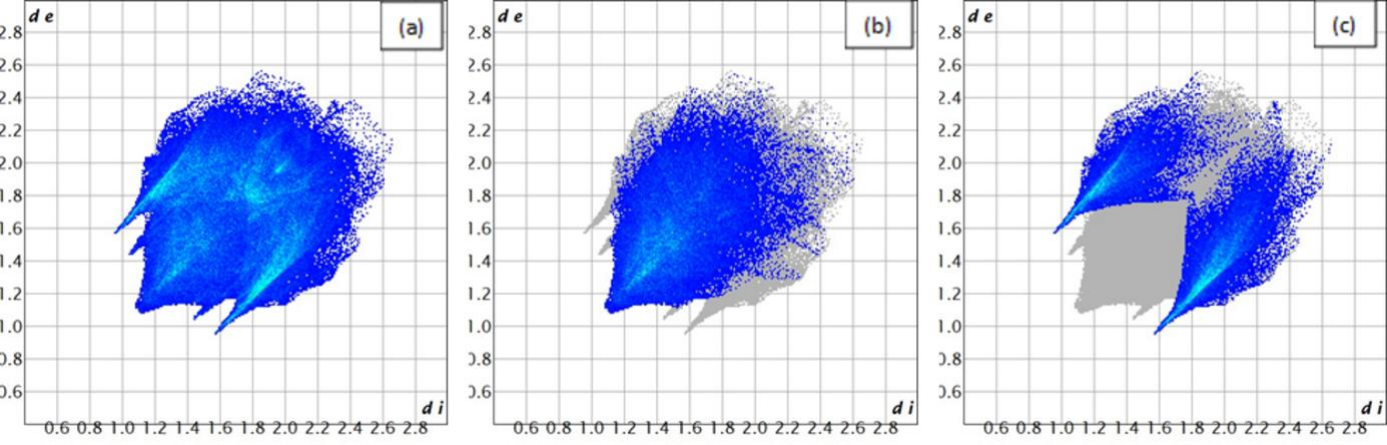
Two-dimensional fingerprint plots for the title compound showing (*a*) all inter­actions and delineated into (*b*) H⋯H (39.9%) and (*c*) H⋯Cl/Cl⋯H (28.2%) contacts. The values *d*_i_ (*x*-axis) and *d*_e_ (*y*-axis) are the closest inter­nal and external distances from given places on the Hirshfeld surface (in Å).

The two-dimensional fingerprint plots of the most abundant contacts are presented in Fig. 3 ▸ and indicate that H⋯H (39.9%) and H⋯Cl/Cl⋯H (28.2%) contacts are responsible for the largest contributions to the Hirshfeld surface. Besides these contacts, C⋯H/H⋯C (7.2%), H⋯N/N⋯H (6.8%) and H⋯O/O⋯H (6.2%) inter­actions also contribute to the total Hirshfeld surface. The contributions of further contacts are only minor and amount to C⋯N/N⋯C (3.8%), C⋯C (3.6%), C⋯O/O⋯C (1.6%), H⋯Zn/Zn⋯H (1.2%), O⋯O (0.3%), N⋯O/O⋯N (0.3%), N⋯Cl/Cl⋯N (0.3%), O⋯Cl/Cl⋯O (0.3%), and C⋯Cl/Cl⋯C (0.3%).

## 4. Synthesis and crystallization

**Figure 4:**
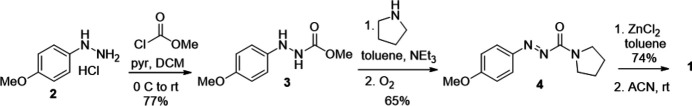
Synthesis of the pyrrolidinyl-4-meth­oxy­phenyl­azoformamide ligand from phenyl­hydrazine hydro­chloride.

The pyrrolidinyl-4-meth­oxy­phenyl­azoformamide ligand was prepared in a two-step synthesis from commercially available 4-meth­oxy­phenyl­hydrazine·HCl and methyl chloro­formate as shown in Fig. 4 ▸. The inter­mediate ester was isolated prior to forming the formamide similar to a recently reported synthesis of biologically active aryl­azo­thio­formamides (Pradhan *et al.*, 2023 ▸).

**Pyrrolidinyl-4-meth­oxy­phenyl­azoformamide (4):** 4-meth­oxy­phenyl­hydrazine·HCl (5.00 mmol, 0.873 g) was dissolved in 20 mL of di­chloro­methane in a round-bottom flask fitted with a magnetic stirrer followed by degassing under nitro­gen flow. Then, pre-dried pyridine (10.0 mmol, 0.805 mL) was added to the solution followed by a dropwise addition of methyl chloro­formate (5.5 mmol, 0.425 mL). The reaction was stirred for 30 minutes at 273 K and 1 h at room temperature. The mixture was diluted with 20 mL of water and was extracted with ether (3 × 40 mL), before the organic layer was separated and concentrated *in vacuo*. Pure product was obtained from column chromatography (3:2 hexa­ne: ethyl acetate) yielding the ester as a light brown solid (0.892 g, 77% yield) identified as methyl 2-(4-meth­oxy­phen­yl)hydrazine-1-carboxyl­ate and matching previously reported NMR data (Käsnänen *et al.*, 2013 ▸). This ester, **3** (4 mmol, 0.785 g), was dissolved in 10 mL of toluene and to the solution was added pyrrolidine (4.8 mmol, 0.473 mL) followed by tri­ethyl­amine (6.0 mmol, 0.855 mL). The solution was refluxed at 363 K under nitro­gen for 48 h. The solution was then cooled to room temperature, opened to air, and stirred for 4 h. The solution was then washed with brine (2 × 25 mL), extracted with ethyl acetate, and dried with MgSO_4_. After concentration, the crude product was subjected to column chromatography (7:3 hexa­ne: ethyl acetate) to give 0.612 g of a bright-orange solid (65% yield). ^1^H NMR (500 MHz, Chloro­form-*d*) δ 7.93 (*d*, *J* = 9.1 Hz, 2H), 6.98 (*d*, *J* = 9.1 Hz, 2H), 3.89 (*s*, 3H), 3.71–3.68 (*m*, 2H), 3.64 (*t*, *J* = 6.8 Hz, 2H), 1.98–1.95 (*m*, 4H). ^13^C NMR (126 MHz, CDCl_3_) δ 161.114, 146.612, 126.022, 114.429, 114.421, 55.801, 46.781, 26.183, 24.470 FTIR (cm^−1^): 2974, 2884, 1690, 1500, 1256, 1025, 848. HRMS [*M* + H]^+^, Measured: 234.1243; found: 234.1236, m.p.339 K.

**Chlorido­bis­{** ***N*** **-[(4-meth­oxy­phen­yl)imino]­pyrrolidine-1-carboxamide}­zinc(II) (aceto­nitrile)­tri­chlorido­zincate (1):** Zinc(II) chloride (0.136 g, 1.00 mmol) was added to a solution of pyrrolidine-4-meth­oxy­phen­ylazoformamide, **4** (0.233 g, 1 mmol) in 5 ml of toluene and the mixture was refluxed for 2 h at 363 K to obtain a yellow solution of the zinc complex. The solution was concentrated by rotary evaporation, and the resulting solid was purified using several cold hexane washes to remove any residual ligand, yielding 0.569 g (74% yield) of a yellow solid. 15–25 mg of the material were dissolved in 2 mL of aceto­nitrile for crystallization. After 2 days of slow evaporation, yellow plate-like crystals were obtained. ^1^H NMR (500 MHz, Chloro­form-*d*) δ 8.04 (*d*, *J* = 8.5 Hz, 4H), 7.04 (*d*, *J* = 7.2 Hz, 4H), 3.92 (*s*, 6H), 3.76 (*dt*, *J* = 12.7, 6.0 Hz, 8H), 2.07–1.98 (*m*, 8H). ^13^C NMR (126 MHz, CDCl_3_) δ 165.150, 161.404, 146.278, 127.316, 114.971, 56.062, 47.320, 26.143, 24.515. FTIR (cm^−1^): 2979, 1647, 1370, 1267, 1047, 846; m.p. 467 K.

## 5. Refinement

Crystal data, data collection and structure refinement details are summarized in Table 1 ▸. The hydrogen atoms were placed in calculated positions with C—H distances of 0.95 Å and refined as riding atoms with *U*_iso_(H) = 1.2*U*_eq_(C). Methyl H atoms were positioned geometrically and were allowed to ride on C atoms and rotate around the C—C bond, with C—H = 0.98 Å and *U*_iso_(H) = 1.5*U*_eq_(C).

## Supplementary Material

Crystal structure: contains datablock(s) I. DOI: 10.1107/S2056989023010447/yy2008sup1.cif

Structure factors: contains datablock(s) I. DOI: 10.1107/S2056989023010447/yy2008Isup2.hkl

Includes NMR data of key precursors and of title compound. DOI: 10.1107/S2056989023010447/yy2008sup3.docx

CCDC reference: 2299802

Additional supporting information:  crystallographic information; 3D view; checkCIF report

## Figures and Tables

**Table 1:** Experimental details

Crystal data	
Chemical formula	[ZnCl(C_12_H_15_N_3_O_2_)_2_][ZnCl_3_(C_2_H_3_N)]
*M*_r_	780.13
Crystal system, space group	Orthorhombic, *P* *b* *c* *a*
Temperature (K)	100
*a*, *b*, *c* (Å)	28.4313 (7), 7.6516 (2), 29.5461 (9)
*V* (Å^3^)	6427.6 (3)
*Z*	8
Radiation type	Mo *K*α
μ (mm^−1^)	1.87
Crystal size (mm)	0.19 × 0.17 × 0.02

Data collection	
Diffractometer	Bruker D8 VENTURE Duo
Absorption correction	Multi-scan (*SADABS*; Krause *et al.*, 2015 ▸)
*T*_min_, *T*_max_	0.665, 0.746
No. of measured, independent and observed [*I* > 2σ(*I*)] reflections	167426, 7354, 5790
*R*_int_	0.088
(sin θ/λ)_max_ (Å^−1^)	0.649

Refinement	
*R*[*F* ^2^ > 2σ(*F* ^2^)], *wR*(*F* ^2^), *S*	0.046, 0.104, 1.15
No. of reflections	7354
No. of parameters	391
H-atom treatment	H-atom parameters constrained
Δρ_max_, Δρ_min_ (e Å^−3^)	1.05, −0.54

Computer programs: *APEX4* and *SAINT* (Bruker, 2021 ▸), *SHELXT2018/2* (Sheldrick, 2015*a* ▸), *SHELXL2018/3* (Sheldrick, 2015*b* ▸), *OLEX2* (Dolomanov *et al.*, 2009 ▸), and Macrae *et al.*, 2020 ▸).

## supplementary crystallographic information

### Crystal data

**Table:**

[ZnCl(C_12_H_15_N_3_O_2_)_2_][ZnCl_3_(C_2_H_3_N)]	*D*_x_ = 1.612 Mg m^−^^3^
*M**_r_* = 780.13	Mo *K*α radiation, λ = 0.71073 Å
Orthorhombic, *P**b**c**a*	Cell parameters from 9702 reflections
*a* = 28.4313 (7) Å	θ = 2.8–27.4°
*b* = 7.6516 (2) Å	µ = 1.87 mm^−^^1^
*c* = 29.5461 (9) Å	*T* = 100 K
*V* = 6427.6 (3) Å^3^	Plate, yellow
*Z* = 8	0.19 × 0.17 × 0.02 mm
*F*(000) = 3184	

### Data collection

**Table:**

Bruker D8 VENTURE Duo diffractometer	7354 independent reflections
Radiation source: sealed tube, fine-focus	5790 reflections with *I* > 2σ(*I*)
TRIUMPH graphite monochromator	*R*_int_ = 0.088
Detector resolution: 7.39 pixels mm^-1^	θ_max_ = 27.5°, θ_min_ = 2.6°
ω and φ scans	*h* = −36→31
Absorption correction: multi-scan (SADABS; Krause *et al.*, 2015)	*k* = −9→9
*T*_min_ = 0.665, *T*_max_ = 0.746	*l* = −38→38
167426 measured reflections	

### Refinement

**Table:**

Refinement on *F*^2^	Primary atom site location: dual
Least-squares matrix: full	Hydrogen site location: inferred from neighbouring sites
*R*[*F*^2^ > 2σ(*F*^2^)] = 0.046	H-atom parameters constrained
*wR*(*F*^2^) = 0.104	*w* = 1/[σ^2^(*F*_o_^2^) + (0.019*P*)^2^ + 31.9684*P*] where *P* = (*F*_o_^2^ + 2*F*_c_^2^)/3
*S* = 1.15	(Δ/σ)_max_ = 0.001
7354 reflections	Δρ_max_ = 1.05 e Å^−^^3^
391 parameters	Δρ_min_ = −0.54 e Å^−^^3^
0 restraints	

### Special details

**Table:**

Geometry. All esds (except the esd in the dihedral angle between two l.s. planes) are estimated using the full covariance matrix. The cell esds are taken into account individually in the estimation of esds in distances, angles and torsion angles; correlations between esds in cell parameters are only used when they are defined by crystal symmetry. An approximate (isotropic) treatment of cell esds is used for estimating esds involving l.s. planes.

### Fractional atomic coordinates and isotropic or equivalent isotropic displacement parameters (Å^2^)

**Table:**

	*x*	*y*	*z*	*U*_iso_*/*U*_eq_	
Zn1	0.38392 (2)	0.32189 (6)	0.53246 (2)	0.01362 (10)	
Cl2	0.35537 (3)	0.06498 (13)	0.51090 (3)	0.01896 (19)	
O1A	0.41294 (9)	0.3767 (4)	0.59256 (9)	0.0187 (6)	
O2A	0.55202 (9)	0.1012 (4)	0.36437 (9)	0.0229 (6)	
N1A	0.46035 (10)	0.2952 (4)	0.52134 (10)	0.0133 (6)	
N2A	0.48565 (10)	0.3201 (4)	0.55606 (10)	0.0154 (6)	
N3A	0.48118 (10)	0.3960 (4)	0.63111 (10)	0.0142 (6)	
C1A	0.45700 (13)	0.3661 (5)	0.59420 (12)	0.0148 (7)	
C2A	0.48421 (13)	0.2478 (5)	0.48157 (12)	0.0155 (7)	
C3A	0.45684 (13)	0.2099 (5)	0.44347 (12)	0.0172 (8)	
H3A	0.423563	0.219067	0.445091	0.021*	
C4A	0.47807 (13)	0.1593 (5)	0.40346 (12)	0.0167 (8)	
H4A	0.459493	0.131954	0.377685	0.020*	
C5A	0.52690 (13)	0.1487 (5)	0.40129 (13)	0.0169 (8)	
C6A	0.55441 (13)	0.1885 (6)	0.43970 (14)	0.0223 (9)	
H6A	0.587724	0.181919	0.437899	0.027*	
C7A	0.53351 (13)	0.2365 (6)	0.47949 (13)	0.0188 (8)	
H7A	0.552075	0.261920	0.505402	0.023*	
C8A	0.45700 (13)	0.4426 (6)	0.67395 (12)	0.0177 (8)	
H8AA	0.430842	0.360891	0.680413	0.021*	
H8AB	0.444552	0.563336	0.672826	0.021*	
C9A	0.49607 (13)	0.4258 (5)	0.70924 (13)	0.0186 (8)	
H9AA	0.497786	0.305092	0.721214	0.022*	
H9AB	0.490918	0.507573	0.734723	0.022*	
C10A	0.54098 (13)	0.4729 (5)	0.68302 (13)	0.0193 (8)	
H10A	0.544762	0.601197	0.680722	0.023*	
H10B	0.569137	0.422980	0.697896	0.023*	
C11A	0.53340 (12)	0.3916 (5)	0.63639 (12)	0.0168 (8)	
H11A	0.549077	0.461079	0.612492	0.020*	
H11B	0.545399	0.270177	0.635372	0.020*	
C12A	0.52662 (14)	0.0592 (6)	0.32387 (13)	0.0218 (9)	
H12A	0.548822	0.024803	0.300113	0.033*	
H12B	0.508739	0.161675	0.313882	0.033*	
H12C	0.504936	−0.037576	0.329936	0.033*	
O1B	0.37393 (9)	0.5185 (4)	0.48827 (9)	0.0157 (5)	
O2B	0.20169 (9)	0.1946 (4)	0.68988 (9)	0.0215 (6)	
N1B	0.31512 (10)	0.4348 (4)	0.55143 (10)	0.0130 (6)	
N2B	0.29743 (11)	0.5328 (4)	0.52118 (10)	0.0139 (6)	
N3B	0.31713 (10)	0.6878 (4)	0.45738 (10)	0.0145 (6)	
C1B	0.33246 (12)	0.5774 (5)	0.48798 (12)	0.0132 (7)	
C2B	0.28426 (13)	0.3787 (5)	0.58569 (12)	0.0134 (7)	
C3B	0.30428 (13)	0.3026 (5)	0.62401 (13)	0.0168 (8)	
H3B	0.337426	0.289260	0.626118	0.020*	
C4B	0.27560 (13)	0.2466 (5)	0.65894 (13)	0.0167 (8)	
H4B	0.289068	0.199929	0.685771	0.020*	
C5B	0.22695 (14)	0.2588 (5)	0.65466 (13)	0.0170 (8)	
C6B	0.20650 (13)	0.3330 (5)	0.61614 (13)	0.0179 (8)	
H6B	0.173273	0.340930	0.613448	0.021*	
C7B	0.23534 (13)	0.3947 (5)	0.58204 (13)	0.0167 (8)	
H7B	0.221930	0.448156	0.556045	0.020*	
C8B	0.34803 (13)	0.7567 (5)	0.42103 (13)	0.0176 (8)	
H8BA	0.357293	0.663033	0.399685	0.021*	
H8BB	0.376717	0.811038	0.433778	0.021*	
C9B	0.31677 (14)	0.8932 (5)	0.39776 (13)	0.0211 (8)	
H9BA	0.321348	1.010256	0.411355	0.025*	
H9BB	0.323686	0.899791	0.364963	0.025*	
C10B	0.26686 (14)	0.8269 (5)	0.40602 (13)	0.0193 (8)	
H10C	0.243535	0.922170	0.402726	0.023*	
H10D	0.258748	0.730994	0.384933	0.023*	
C11B	0.26919 (13)	0.7620 (5)	0.45464 (13)	0.0174 (8)	
H11C	0.265053	0.859231	0.476389	0.021*	
H11D	0.245005	0.671737	0.460494	0.021*	
C12B	0.15132 (13)	0.1884 (6)	0.68501 (14)	0.0245 (9)	
H12D	0.143225	0.125226	0.657227	0.037*	
H12E	0.138870	0.307660	0.683382	0.037*	
H12F	0.137575	0.128065	0.711113	0.037*	
Zn1C	0.36227 (2)	0.31087 (6)	0.79662 (2)	0.01579 (11)	
Cl1C	0.37575 (3)	0.15712 (13)	0.73339 (3)	0.0198 (2)	
Cl2C	0.40356 (3)	0.55957 (13)	0.79884 (3)	0.0216 (2)	
Cl3C	0.36050 (3)	0.17133 (13)	0.86338 (3)	0.0205 (2)	
N1C	0.29085 (12)	0.3713 (5)	0.78907 (12)	0.0232 (8)	
C1C	0.25046 (15)	0.3671 (5)	0.78836 (13)	0.0209 (8)	
C2C	0.19989 (13)	0.3640 (6)	0.78872 (14)	0.0213 (9)	
H2CA	0.187897	0.435302	0.763698	0.032*	
H2CB	0.188409	0.411243	0.817500	0.032*	
H2CC	0.188907	0.243370	0.785197	0.032*	

### Atomic displacement parameters (Å^2^)

**Table:**

	*U*^11^	*U*^22^	*U*^33^	*U*^12^	*U*^13^	*U*^23^
Zn1	0.00912 (19)	0.0189 (2)	0.0128 (2)	−0.00014 (17)	−0.00164 (15)	−0.00036 (18)
Cl2	0.0149 (4)	0.0205 (5)	0.0215 (5)	−0.0035 (4)	0.0028 (4)	−0.0032 (4)
O1A	0.0113 (12)	0.0294 (15)	0.0154 (13)	−0.0016 (11)	−0.0021 (10)	−0.0013 (12)
O2A	0.0149 (13)	0.0378 (17)	0.0160 (14)	0.0033 (12)	0.0018 (11)	−0.0041 (13)
N1A	0.0108 (14)	0.0183 (16)	0.0110 (15)	−0.0008 (12)	−0.0008 (11)	0.0010 (12)
N2A	0.0129 (14)	0.0217 (17)	0.0116 (15)	−0.0002 (13)	−0.0022 (12)	0.0015 (13)
N3A	0.0092 (14)	0.0216 (17)	0.0116 (15)	−0.0005 (12)	0.0012 (12)	0.0010 (13)
C1A	0.0144 (17)	0.0174 (18)	0.0124 (17)	−0.0014 (14)	−0.0002 (14)	0.0019 (14)
C2A	0.0157 (17)	0.0172 (18)	0.0137 (18)	0.0016 (15)	−0.0001 (14)	0.0021 (15)
C3A	0.0128 (17)	0.023 (2)	0.0158 (19)	−0.0017 (15)	0.0006 (14)	0.0000 (16)
C4A	0.0145 (17)	0.023 (2)	0.0124 (17)	−0.0017 (15)	−0.0012 (14)	0.0004 (16)
C5A	0.0176 (18)	0.020 (2)	0.0128 (17)	0.0037 (15)	0.0025 (15)	0.0027 (15)
C6A	0.0086 (16)	0.036 (2)	0.022 (2)	0.0011 (17)	0.0003 (15)	0.0020 (19)
C7A	0.0151 (18)	0.030 (2)	0.0117 (18)	0.0007 (16)	−0.0051 (14)	−0.0007 (16)
C8A	0.0142 (18)	0.026 (2)	0.0130 (18)	−0.0022 (16)	0.0002 (14)	−0.0001 (16)
C9A	0.0190 (19)	0.025 (2)	0.0119 (18)	0.0012 (16)	−0.0030 (15)	−0.0002 (16)
C10A	0.0148 (18)	0.023 (2)	0.020 (2)	−0.0004 (16)	−0.0046 (15)	−0.0007 (16)
C11A	0.0115 (17)	0.026 (2)	0.0126 (18)	0.0001 (15)	−0.0032 (14)	0.0025 (16)
C12A	0.024 (2)	0.027 (2)	0.0146 (19)	0.0010 (18)	0.0008 (16)	−0.0061 (17)
O1B	0.0103 (12)	0.0208 (14)	0.0162 (13)	0.0003 (10)	0.0012 (10)	0.0032 (11)
O2B	0.0159 (13)	0.0321 (16)	0.0164 (13)	−0.0041 (12)	0.0037 (11)	0.0020 (12)
N1B	0.0126 (14)	0.0149 (15)	0.0115 (15)	−0.0017 (12)	−0.0023 (12)	−0.0016 (12)
N2B	0.0130 (14)	0.0172 (16)	0.0115 (15)	−0.0032 (12)	0.0012 (12)	0.0012 (12)
N3B	0.0133 (14)	0.0170 (15)	0.0131 (15)	−0.0007 (13)	0.0008 (12)	0.0016 (13)
C1B	0.0118 (17)	0.0161 (18)	0.0118 (17)	−0.0026 (14)	0.0000 (13)	−0.0027 (14)
C2B	0.0155 (17)	0.0143 (17)	0.0105 (17)	−0.0002 (14)	0.0023 (14)	−0.0022 (14)
C3B	0.0108 (16)	0.021 (2)	0.0184 (19)	0.0008 (15)	−0.0009 (14)	0.0005 (16)
C4B	0.0182 (18)	0.0217 (19)	0.0101 (17)	−0.0004 (16)	−0.0002 (14)	−0.0003 (15)
C5B	0.0219 (19)	0.0193 (19)	0.0098 (17)	−0.0024 (16)	0.0015 (15)	−0.0038 (15)
C6B	0.0107 (16)	0.023 (2)	0.0198 (19)	−0.0008 (15)	0.0005 (14)	−0.0040 (17)
C7B	0.0153 (17)	0.021 (2)	0.0140 (18)	0.0001 (15)	−0.0018 (14)	−0.0011 (15)
C8B	0.0168 (18)	0.0205 (19)	0.0153 (18)	−0.0012 (15)	0.0034 (15)	0.0025 (15)
C9B	0.030 (2)	0.020 (2)	0.0137 (19)	−0.0012 (17)	0.0000 (16)	0.0037 (16)
C10B	0.0230 (19)	0.0192 (19)	0.0157 (18)	0.0027 (16)	−0.0045 (15)	0.0023 (16)
C11B	0.0153 (18)	0.0179 (19)	0.0189 (19)	0.0034 (15)	−0.0041 (15)	0.0018 (15)
C12B	0.0146 (18)	0.039 (3)	0.020 (2)	−0.0044 (18)	0.0067 (15)	0.0000 (19)
Zn1C	0.0128 (2)	0.0178 (2)	0.0167 (2)	0.00073 (17)	0.00144 (16)	0.00044 (18)
Cl1C	0.0193 (4)	0.0226 (5)	0.0174 (4)	0.0003 (4)	0.0029 (4)	−0.0003 (4)
Cl2C	0.0193 (4)	0.0212 (5)	0.0244 (5)	−0.0042 (4)	−0.0017 (4)	0.0005 (4)
Cl3C	0.0222 (4)	0.0211 (5)	0.0180 (4)	0.0010 (4)	0.0017 (4)	0.0022 (4)
N1C	0.0196 (18)	0.031 (2)	0.0189 (18)	0.0049 (15)	−0.0022 (14)	−0.0016 (15)
C1C	0.021 (2)	0.020 (2)	0.021 (2)	0.0044 (16)	0.0020 (17)	−0.0002 (16)
C2C	0.0155 (19)	0.027 (2)	0.022 (2)	0.0029 (16)	−0.0005 (16)	0.0000 (17)

### Geometric parameters (Å, º)

**Table:**

Zn1—Cl2	2.2202 (10)	O2B—C12B	1.440 (5)
Zn1—O1A	2.002 (3)	N1B—N2B	1.270 (4)
Zn1—N1A	2.207 (3)	N1B—C2B	1.407 (5)
Zn1—O1B	2.012 (3)	N2B—C1B	1.439 (5)
Zn1—N1B	2.211 (3)	N3B—C1B	1.311 (5)
O1A—C1A	1.256 (4)	N3B—C8B	1.484 (5)
O2A—C5A	1.354 (4)	N3B—C11B	1.479 (5)
O2A—C12A	1.434 (5)	C2B—C3B	1.395 (5)
N1A—N2A	1.267 (4)	C2B—C7B	1.400 (5)
N1A—C2A	1.405 (5)	C3B—H3B	0.9500
N2A—C1A	1.435 (5)	C3B—C4B	1.383 (5)
N3A—C1A	1.309 (5)	C4B—H4B	0.9500
N3A—C8A	1.484 (5)	C4B—C5B	1.392 (5)
N3A—C11A	1.493 (4)	C5B—C6B	1.399 (5)
C2A—C3A	1.399 (5)	C6B—H6B	0.9500
C2A—C7A	1.406 (5)	C6B—C7B	1.382 (5)
C3A—H3A	0.9500	C7B—H7B	0.9500
C3A—C4A	1.383 (5)	C8B—H8BA	0.9900
C4A—H4A	0.9500	C8B—H8BB	0.9900
C4A—C5A	1.392 (5)	C8B—C9B	1.535 (5)
C5A—C6A	1.412 (5)	C9B—H9BA	0.9900
C6A—H6A	0.9500	C9B—H9BB	0.9900
C6A—C7A	1.367 (6)	C9B—C10B	1.527 (6)
C7A—H7A	0.9500	C10B—H10C	0.9900
C8A—H8AA	0.9900	C10B—H10D	0.9900
C8A—H8AB	0.9900	C10B—C11B	1.521 (5)
C8A—C9A	1.529 (5)	C11B—H11C	0.9900
C9A—H9AA	0.9900	C11B—H11D	0.9900
C9A—H9AB	0.9900	C12B—H12D	0.9800
C9A—C10A	1.536 (5)	C12B—H12E	0.9800
C10A—H10A	0.9900	C12B—H12F	0.9800
C10A—H10B	0.9900	Zn1C—Cl1C	2.2408 (10)
C10A—C11A	1.527 (5)	Zn1C—Cl2C	2.2368 (11)
C11A—H11A	0.9900	Zn1C—Cl3C	2.2435 (10)
C11A—H11B	0.9900	Zn1C—N1C	2.095 (3)
C12A—H12A	0.9800	N1C—C1C	1.149 (5)
C12A—H12B	0.9800	C1C—C2C	1.438 (5)
C12A—H12C	0.9800	C2C—H2CA	0.9800
O1B—C1B	1.262 (4)	C2C—H2CB	0.9800
O2B—C5B	1.356 (5)	C2C—H2CC	0.9800
			
O1A—Zn1—Cl2	126.20 (9)	N2B—N1B—Zn1	113.7 (2)
O1A—Zn1—N1A	75.27 (11)	N2B—N1B—C2B	116.1 (3)
O1A—Zn1—O1B	118.48 (11)	C2B—N1B—Zn1	128.0 (2)
O1A—Zn1—N1B	93.31 (11)	N1B—N2B—C1B	110.2 (3)
N1A—Zn1—Cl2	103.62 (9)	C1B—N3B—C8B	122.1 (3)
N1A—Zn1—N1B	160.92 (12)	C1B—N3B—C11B	126.3 (3)
O1B—Zn1—Cl2	115.10 (8)	C11B—N3B—C8B	111.7 (3)
O1B—Zn1—N1A	96.41 (11)	O1B—C1B—N2B	123.9 (3)
O1B—Zn1—N1B	75.36 (11)	O1B—C1B—N3B	123.0 (3)
N1B—Zn1—Cl2	95.47 (8)	N3B—C1B—N2B	113.1 (3)
C1A—O1A—Zn1	115.6 (2)	C3B—C2B—N1B	117.2 (3)
C5A—O2A—C12A	117.8 (3)	C3B—C2B—C7B	120.3 (3)
N2A—N1A—Zn1	115.1 (2)	C7B—C2B—N1B	122.5 (3)
N2A—N1A—C2A	116.2 (3)	C2B—C3B—H3B	120.2
C2A—N1A—Zn1	128.6 (2)	C4B—C3B—C2B	119.6 (3)
N1A—N2A—C1A	110.5 (3)	C4B—C3B—H3B	120.2
C1A—N3A—C8A	120.6 (3)	C3B—C4B—H4B	120.1
C1A—N3A—C11A	127.2 (3)	C3B—C4B—C5B	119.8 (4)
C8A—N3A—C11A	112.1 (3)	C5B—C4B—H4B	120.1
O1A—C1A—N2A	123.5 (3)	O2B—C5B—C4B	115.6 (4)
O1A—C1A—N3A	123.0 (3)	O2B—C5B—C6B	123.5 (3)
N3A—C1A—N2A	113.5 (3)	C4B—C5B—C6B	120.9 (4)
N1A—C2A—C7A	122.3 (3)	C5B—C6B—H6B	120.5
C3A—C2A—N1A	117.3 (3)	C7B—C6B—C5B	119.0 (3)
C3A—C2A—C7A	120.5 (4)	C7B—C6B—H6B	120.5
C2A—C3A—H3A	119.9	C2B—C7B—H7B	119.9
C4A—C3A—C2A	120.2 (3)	C6B—C7B—C2B	120.2 (4)
C4A—C3A—H3A	119.9	C6B—C7B—H7B	119.9
C3A—C4A—H4A	120.3	N3B—C8B—H8BA	111.2
C3A—C4A—C5A	119.4 (4)	N3B—C8B—H8BB	111.2
C5A—C4A—H4A	120.3	N3B—C8B—C9B	102.9 (3)
O2A—C5A—C4A	125.4 (4)	H8BA—C8B—H8BB	109.1
O2A—C5A—C6A	114.4 (3)	C9B—C8B—H8BA	111.2
C4A—C5A—C6A	120.2 (4)	C9B—C8B—H8BB	111.2
C5A—C6A—H6A	119.7	C8B—C9B—H9BA	111.0
C7A—C6A—C5A	120.6 (3)	C8B—C9B—H9BB	111.0
C7A—C6A—H6A	119.7	H9BA—C9B—H9BB	109.0
C2A—C7A—H7A	120.4	C10B—C9B—C8B	103.9 (3)
C6A—C7A—C2A	119.2 (4)	C10B—C9B—H9BA	111.0
C6A—C7A—H7A	120.4	C10B—C9B—H9BB	111.0
N3A—C8A—H8AA	111.2	C9B—C10B—H10C	111.2
N3A—C8A—H8AB	111.2	C9B—C10B—H10D	111.2
N3A—C8A—C9A	103.0 (3)	H10C—C10B—H10D	109.2
H8AA—C8A—H8AB	109.1	C11B—C10B—C9B	102.6 (3)
C9A—C8A—H8AA	111.2	C11B—C10B—H10C	111.2
C9A—C8A—H8AB	111.2	C11B—C10B—H10D	111.2
C8A—C9A—H9AA	111.0	N3B—C11B—C10B	102.6 (3)
C8A—C9A—H9AB	111.0	N3B—C11B—H11C	111.3
C8A—C9A—C10A	103.9 (3)	N3B—C11B—H11D	111.3
H9AA—C9A—H9AB	109.0	C10B—C11B—H11C	111.3
C10A—C9A—H9AA	111.0	C10B—C11B—H11D	111.3
C10A—C9A—H9AB	111.0	H11C—C11B—H11D	109.2
C9A—C10A—H10A	111.0	O2B—C12B—H12D	109.5
C9A—C10A—H10B	111.0	O2B—C12B—H12E	109.5
H10A—C10A—H10B	109.0	O2B—C12B—H12F	109.5
C11A—C10A—C9A	104.0 (3)	H12D—C12B—H12E	109.5
C11A—C10A—H10A	111.0	H12D—C12B—H12F	109.5
C11A—C10A—H10B	111.0	H12E—C12B—H12F	109.5
N3A—C11A—C10A	103.0 (3)	Cl1C—Zn1C—Cl3C	119.15 (4)
N3A—C11A—H11A	111.2	Cl2C—Zn1C—Cl1C	112.42 (4)
N3A—C11A—H11B	111.2	Cl2C—Zn1C—Cl3C	113.01 (4)
C10A—C11A—H11A	111.2	N1C—Zn1C—Cl1C	101.12 (10)
C10A—C11A—H11B	111.2	N1C—Zn1C—Cl2C	108.91 (11)
H11A—C11A—H11B	109.1	N1C—Zn1C—Cl3C	100.19 (10)
O2A—C12A—H12A	109.5	C1C—N1C—Zn1C	164.7 (4)
O2A—C12A—H12B	109.5	N1C—C1C—C2C	178.4 (5)
O2A—C12A—H12C	109.5	C1C—C2C—H2CA	109.5
H12A—C12A—H12B	109.5	C1C—C2C—H2CB	109.5
H12A—C12A—H12C	109.5	C1C—C2C—H2CC	109.5
H12B—C12A—H12C	109.5	H2CA—C2C—H2CB	109.5
C1B—O1B—Zn1	113.8 (2)	H2CA—C2C—H2CC	109.5
C5B—O2B—C12B	117.5 (3)	H2CB—C2C—H2CC	109.5
			
Zn1—O1A—C1A—N2A	0.7 (5)	C11A—N3A—C1A—O1A	−178.3 (4)
Zn1—O1A—C1A—N3A	−179.2 (3)	C11A—N3A—C1A—N2A	1.8 (6)
Zn1—N1A—N2A—C1A	−2.0 (4)	C11A—N3A—C8A—C9A	−12.7 (4)
Zn1—N1A—C2A—C3A	−0.7 (5)	C12A—O2A—C5A—C4A	0.5 (6)
Zn1—N1A—C2A—C7A	179.8 (3)	C12A—O2A—C5A—C6A	−179.8 (4)
Zn1—O1B—C1B—N2B	10.7 (4)	O2B—C5B—C6B—C7B	−180.0 (4)
Zn1—O1B—C1B—N3B	−170.1 (3)	N1B—N2B—C1B—O1B	3.5 (5)
Zn1—N1B—N2B—C1B	−14.5 (4)	N1B—N2B—C1B—N3B	−175.8 (3)
Zn1—N1B—C2B—C3B	31.2 (5)	N1B—C2B—C3B—C4B	179.4 (3)
Zn1—N1B—C2B—C7B	−147.8 (3)	N1B—C2B—C7B—C6B	178.2 (3)
O2A—C5A—C6A—C7A	−179.3 (4)	N2B—N1B—C2B—C3B	−166.9 (3)
N1A—N2A—C1A—O1A	1.0 (5)	N2B—N1B—C2B—C7B	14.0 (5)
N1A—N2A—C1A—N3A	−179.1 (3)	N3B—C8B—C9B—C10B	−27.7 (4)
N1A—C2A—C3A—C4A	−178.8 (4)	C1B—N3B—C8B—C9B	−173.7 (3)
N1A—C2A—C7A—C6A	179.6 (4)	C1B—N3B—C11B—C10B	−161.6 (4)
N2A—N1A—C2A—C3A	176.5 (3)	C2B—N1B—N2B—C1B	−178.9 (3)
N2A—N1A—C2A—C7A	−3.0 (6)	C2B—C3B—C4B—C5B	3.1 (6)
N3A—C8A—C9A—C10A	30.7 (4)	C3B—C2B—C7B—C6B	−0.9 (6)
C1A—N3A—C8A—C9A	168.0 (3)	C3B—C4B—C5B—O2B	177.6 (4)
C1A—N3A—C11A—C10A	168.6 (4)	C3B—C4B—C5B—C6B	−2.4 (6)
C2A—N1A—N2A—C1A	−179.5 (3)	C4B—C5B—C6B—C7B	0.0 (6)
C2A—C3A—C4A—C5A	−0.9 (6)	C5B—C6B—C7B—C2B	1.6 (6)
C3A—C2A—C7A—C6A	0.2 (6)	C7B—C2B—C3B—C4B	−1.5 (6)
C3A—C4A—C5A—O2A	−179.9 (4)	C8B—N3B—C1B—O1B	−1.8 (6)
C3A—C4A—C5A—C6A	0.3 (6)	C8B—N3B—C1B—N2B	177.6 (3)
C4A—C5A—C6A—C7A	0.5 (6)	C8B—N3B—C11B—C10B	19.8 (4)
C5A—C6A—C7A—C2A	−0.8 (7)	C8B—C9B—C10B—C11B	40.2 (4)
C7A—C2A—C3A—C4A	0.7 (6)	C9B—C10B—C11B—N3B	−36.3 (4)
C8A—N3A—C1A—O1A	0.9 (6)	C11B—N3B—C1B—O1B	179.7 (3)
C8A—N3A—C1A—N2A	−179.0 (3)	C11B—N3B—C1B—N2B	−0.9 (5)
C8A—N3A—C11A—C10A	−10.6 (4)	C11B—N3B—C8B—C9B	4.9 (4)
C8A—C9A—C10A—C11A	−38.0 (4)	C12B—O2B—C5B—C4B	−173.9 (4)
C9A—C10A—C11A—N3A	29.5 (4)	C12B—O2B—C5B—C6B	6.1 (6)
