# Supplementary material for: The synthesis and structural properties of a chlorido­bis­{N-[(4-meth­oxy­phen­yl)imino]­pyrrolidine-1-carboxamide}­zinc(II) (aceto­nitrile)­trichlorido­zincate coordination complex
Source: Acta Crystallogr E Crystallogr Commun. 2024 Jan 1;80(Pt 1):14–7. doi: 10.1107/S2056989023010447 (PMC10833373; doi:10.1107/S2056989023010447)
Supplement: Supplementary file 3 [file e-80-00014-sup3.docx]

**Supporting Information**

**The synthesis and structural properties of a chlorozinc(II) bis(pyrrolidinyl-4-methoxyphenylazoformamido) acetonitriletrichlorozincate coordination complex**

Laxmi Tiwari^a^ and [Kristopher V. Waynant](https://scripts.iucr.org/cgi-bin/citedin?search_on=name&author_name=Waynant%2C%20K%2EV%2E)^a^

^a^Department of Chemistry, University of Idaho, 875 Perimeter Dr. MS 2343 Moscow, ID 83844

**Contents**

1. **Crystallographic data**
2. **NMR spectra**

**1.Crystallographic data**

**Crystal data**

| C_26_H_33_Cl_4_N_7_O_4_Zn_2_ | *F*(000) = 3184 |
| --- | --- |
| *M_r_* = 780.13 | *D*_x_ = 1.612Mg m^−3^ |
| Orthorhombic, $Pbca$ (61) | Mo *K*α radiation, λ = 0.71073 Å |
| *a* = 28.4313(7)Å | Cell parameters from 4249 reflections |
| *b* = 7.6516(2)Å | 2θ = 5.11 to 54.98 ° |
| *c* = 29.5461(9)Å | µ = 1.869mm^−1^ |
| β = 90° | *T* = 100 K |
| *V* = 6427.6(3)Å^3^ | Plate, yellow |
| *Z* = 8 | 0.02×0.17×0.19 mm |

| Bruker D8 VENTURE Duo Fixed Chi Three-Circle Diffractometer | 7354 independent reflections |
| --- | --- |
| Radiation source: sealed X-ray tube, | 5790 reflections with *I* > 2σ(*I*) |
| TRIUMPH graphite monochromator | *R*_int_ = 0.0881 |
| ω and f scans | θ_max_ = 25.2°, θ_min_ = 2.5° |
| Absorption correction: multi-scan (SADABS; Bruker, 2016) | *h* = −36→31 |
| *T*_min_ = 0.6653, *T*_max_ = 0.7456 | *k* = −9→9 |
| 167426 measured reflections | *l* = −38→38 |

**Data collection**

**Refinement**

| Refinement on *F*^2^ | Primary atom site location: dual |
| --- | --- |
| Least-squares matrix: full | Secondary atom site location: difference Fourier map |
| *R*[*F*^2^ > 2σ(*F*^2^)] = 0.0673 | Hydrogen site location: inferred from neighbouring sites |
| *wR*(*F*^2^) = 0.1039 | H-atom parameters constrained |
| *S* = 1.15 | *w* = 1/[σ^2^(*F*_o_^2^) + (0.0190*P*)^2^ + 31.9684*P*] where *P* = (*F*_o_^2^ + 2*F*_c_^2^)/3 |
| 7354 reflections | (Δ/σ)_max_ < 0.001 |
| 391 parameters | Δρ_max_ = 1.05e Å^−3^ |
| 0 restraints | Δρ_min_ = −0.54 e Å^−3^ |

**Special details**

**Geometry**.  All esds (except the esd in the dihedral angle between two l.s. planes) are estimated using the full covariance matrix. The cell esds are considered individually in the estimation of esds in distances, angles and torsion angles; correlations between esds in cell parameters are only used when they are defined by crystal symmetry. An approximate (isotropic) treatment of cell esds is used for estimating esds involving l.s. planes.

## Fractional atomic coordinates and isotropic or equivalent isotropic displacement parameters (Å^2^)

| **Atom** | ***x*** | ***y*** | ***z*** | ***U*_eq_** |
| --- | --- | --- | --- | --- |
| Zn1 | 0.38392(2) | 0.32189(6) | 0.53246(2) | 0.01362(10) |
| Cl2 | 0.35537(3) | 0.06498(13) | 0.51090(3) | 0.01896(19) |
| O1A | 0.41294(9) | 0.3767(4) | 0.59256(9) | 0.0187(6) |
| O2A | 0.55202(9) | 0.1012(4) | 0.36437(9) | 0.0229(6) |
| N1A | 0.46035(10) | 0.2952(4) | 0.52134(10) | 0.0133(6) |
| N2A | 0.48565(10) | 0.3201(4) | 0.55606(10) | 0.0154(6) |
| N3A | 0.48118(10) | 0.3960(4) | 0.63111(10) | 0.0142(6) |
| C1A | 0.45700(13) | 0.3661(5) | 0.59420(12) | 0.0148(7) |
| C2A | 0.48421(13) | 0.2478(5) | 0.48157(12) | 0.0155(7) |
| C3A | 0.45684(13) | 0.2099(5) | 0.44347(12) | 0.0172(8) |
| H3A | 0.423563 | 0.219067 | 0.445091 | 0.021 |
| C4A | 0.47807(13) | 0.1593(5) | 0.40346(12) | 0.0167(8) |
| H4A | 0.459493 | 0.131954 | 0.377685 | 0.020 |
| C5A | 0.52690(13) | 0.1487(5) | 0.40129(13) | 0.0169(8) |
| C6A | 0.55441(13) | 0.1885(6) | 0.43970(14) | 0.0223(9) |
| H6A | 0.587724 | 0.181919 | 0.437899 | 0.027 |
| C7A | 0.53351(13) | 0.2365(6) | 0.47949(13) | 0.0188(8) |
| H7A | 0.552075 | 0.261920 | 0.505402 | 0.023 |
| C8A | 0.45700(13) | 0.4426(6) | 0.67395(12) | 0.0177(8) |
| H8AA | 0.430842 | 0.360891 | 0.680413 | 0.021 |
| H8AB | 0.444552 | 0.563336 | 0.672826 | 0.021 |
| C9A | 0.49607(13) | 0.4258(5) | 0.70924(13) | 0.0186(8) |
| H9AA | 0.497786 | 0.305092 | 0.721214 | 0.022 |
| H9AB | 0.490918 | 0.507573 | 0.734723 | 0.022 |
| C10A | 0.54098(13) | 0.4729(5) | 0.68302(13) | 0.0193(8) |
| H10A | 0.544762 | 0.601197 | 0.680722 | 0.023 |
| H10B | 0.569137 | 0.422980 | 0.697896 | 0.023 |
| C11A | 0.53340(12) | 0.3916(5) | 0.63639(12) | 0.0168(8) |
| H11A | 0.549077 | 0.461079 | 0.612492 | 0.020 |
| H11B | 0.545399 | 0.270177 | 0.635372 | 0.020 |
| C12A | 0.52662(14) | 0.0592(6) | 0.32387(13) | 0.0218(9) |
| H12A | 0.548822 | 0.024803 | 0.300113 | 0.033 |
| H12B | 0.508739 | 0.161675 | 0.313882 | 0.033 |
| H12C | 0.504936 | −0.037576 | 0.329936 | 0.033 |
| O1B | 0.37393(9) | 0.5185(4) | 0.48827(9) | 0.0157(5) |
| O2B | 0.20169(9) | 0.1946(4) | 0.68988(9) | 0.0215(6) |
| N1B | 0.31512(10) | 0.4348(4) | 0.55143(10) | 0.0130(6) |
| N2B | 0.29743(11) | 0.5328(4) | 0.52118(10) | 0.0139(6) |
| N3B | 0.31713(10) | 0.6878(4) | 0.45738(10) | 0.0145(6) |
| C1B | 0.33246(12) | 0.5774(5) | 0.48798(12) | 0.0132(7) |
| C2B | 0.28426(13) | 0.3787(5) | 0.58569(12) | 0.0134(7) |
| C3B | 0.30428(13) | 0.3026(5) | 0.62401(13) | 0.0168(8) |
| H3B | 0.337426 | 0.289260 | 0.626118 | 0.020 |
| C4B | 0.27560(13) | 0.2466(5) | 0.65894(13) | 0.0167(8) |
| H4B | 0.289068 | 0.199929 | 0.685771 | 0.020 |
| C5B | 0.22695(14) | 0.2588(5) | 0.65466(13) | 0.0170(8) |
| C6B | 0.20650(13) | 0.3330(5) | 0.61614(13) | 0.0179(8) |
| H6B | 0.173273 | 0.340930 | 0.613448 | 0.021 |
| C7B | 0.23534(13) | 0.3947(5) | 0.58204(13) | 0.0167(8) |
| H7B | 0.221930 | 0.448156 | 0.556045 | 0.020 |
| C8B | 0.34803(13) | 0.7567(5) | 0.42103(13) | 0.0176(8) |
| H8BA | 0.357293 | 0.663033 | 0.399685 | 0.021 |
| H8BB | 0.376717 | 0.811038 | 0.433778 | 0.021 |
| C9B | 0.31677(14) | 0.8932(5) | 0.39776(13) | 0.0211(8) |
| H9BA | 0.321348 | 1.010256 | 0.411355 | 0.025 |
| H9BB | 0.323686 | 0.899791 | 0.364963 | 0.025 |
| C10B | 0.26686(14) | 0.8269(5) | 0.40602(13) | 0.0193(8) |
| H10C | 0.243535 | 0.922170 | 0.402726 | 0.023 |
| H10D | 0.258748 | 0.730994 | 0.384933 | 0.023 |
| C11B | 0.26919(13) | 0.7620(5) | 0.45464(13) | 0.0174(8) |
| H11C | 0.265053 | 0.859231 | 0.476389 | 0.021 |
| H11D | 0.245005 | 0.671737 | 0.460494 | 0.021 |
| C12B | 0.15132(13) | 0.1884(6) | 0.68501(14) | 0.0245(9) |
| H12D | 0.143225 | 0.125226 | 0.657227 | 0.037 |
| H12E | 0.138870 | 0.307660 | 0.683382 | 0.037 |
| H12F | 0.137575 | 0.128065 | 0.711113 | 0.037 |
| Zn1C | 0.36227(2) | 0.31087(6) | 0.79662(2) | 0.01579(11) |
| Cl1C | 0.37575(3) | 0.15712(13) | 0.73339(3) | 0.0198(2) |
| Cl2C | 0.40356(3) | 0.55957(13) | 0.79884(3) | 0.0216(2) |
| Cl3C | 0.36050(3) | 0.17133(13) | 0.86338(3) | 0.0205(2) |
| N1C | 0.29085(12) | 0.3713(5) | 0.78907(12) | 0.0232(8) |
| C1C | 0.25046(15) | 0.3671(5) | 0.78836(13) | 0.0209(8) |
| C2C | 0.19989(13) | 0.3640(6) | 0.78872(14) | 0.0213(9) |
| H2CA | 0.187897 | 0.435302 | 0.763698 | 0.032 |
| H2CB | 0.188409 | 0.411243 | 0.817500 | 0.032 |
| H2CC | 0.188907 | 0.243370 | 0.785197 | 0.032 |

*U*_eq_ is defined as 1/3 of the trace of the orthogonalized *U_ij_* tensor.

**Anisotropic displacement parameters (Å^2^)**

| **Atom** | ***U*_11_** | ***U*_22_** | ***U*_33_** | ***U*_23_** | ***U*_13_** | ***U*_12_** |
| --- | --- | --- | --- | --- | --- | --- |
| Zn1 | 0.00912(19) | 0.0189(2) | 0.0128(2) | −0.00036(18) | −0.00164(15) | −0.00014(17) |
| Cl2 | 0.0149(4) | 0.0205(5) | 0.0215(5) | −0.0032(4) | 0.0028(4) | −0.0035(4) |
| O1A | 0.0113(12) | 0.0294(15) | 0.0154(13) | −0.0013(12) | −0.0021(10) | −0.0016(11) |
| O2A | 0.0149(13) | 0.0378(17) | 0.0160(14) | −0.0041(13) | 0.0018(11) | 0.0033(12) |
| N1A | 0.0108(14) | 0.0183(16) | 0.0110(15) | 0.0010(12) | −0.0008(11) | −0.0008(12) |
| N2A | 0.0129(14) | 0.0217(17) | 0.0116(15) | 0.0015(13) | −0.0022(12) | −0.0002(13) |
| N3A | 0.0092(14) | 0.0216(17) | 0.0116(15) | 0.0010(13) | 0.0012(12) | −0.0005(12) |
| C1A | 0.0144(17) | 0.0174(18) | 0.0124(17) | 0.0019(14) | −0.0002(14) | −0.0014(14) |
| C2A | 0.0157(17) | 0.0172(18) | 0.0137(18) | 0.0021(15) | −0.0001(14) | 0.0016(15) |
| C3A | 0.0128(17) | 0.023(2) | 0.0158(19) | 0.0000(16) | 0.0006(14) | −0.0017(15) |
| C4A | 0.0145(17) | 0.023(2) | 0.0124(17) | 0.0004(16) | −0.0012(14) | −0.0017(15) |
| C5A | 0.0176(18) | 0.020(2) | 0.0128(17) | 0.0027(15) | 0.0025(15) | 0.0037(15) |
| C6A | 0.0086(16) | 0.036(2) | 0.022(2) | 0.0020(19) | 0.0003(15) | 0.0011(17) |
| C7A | 0.0151(18) | 0.030(2) | 0.0117(18) | −0.0007(16) | −0.0051(14) | 0.0007(16) |
| C8A | 0.0142(18) | 0.026(2) | 0.0130(18) | −0.0001(16) | 0.0002(14) | −0.0022(16) |
| C9A | 0.0190(19) | 0.025(2) | 0.0119(18) | −0.0002(16) | −0.0030(15) | 0.0012(16) |
| C10A | 0.0148(18) | 0.023(2) | 0.020(2) | −0.0007(16) | −0.0046(15) | −0.0004(16) |
| C11A | 0.0115(17) | 0.026(2) | 0.0126(18) | 0.0025(16) | −0.0032(14) | 0.0001(15) |
| C12A | 0.024(2) | 0.027(2) | 0.0146(19) | −0.0061(17) | 0.0008(16) | 0.0010(18) |
| O1B | 0.0103(12) | 0.0208(14) | 0.0162(13) | 0.0032(11) | 0.0012(10) | 0.0003(10) |
| O2B | 0.0159(13) | 0.0321(16) | 0.0164(13) | 0.0020(12) | 0.0037(11) | −0.0041(12) |
| N1B | 0.0126(14) | 0.0149(15) | 0.0115(15) | −0.0016(12) | −0.0023(12) | −0.0017(12) |
| N2B | 0.0130(14) | 0.0172(16) | 0.0115(15) | 0.0012(12) | 0.0012(12) | −0.0032(12) |
| N3B | 0.0133(14) | 0.0170(15) | 0.0131(15) | 0.0016(13) | 0.0008(12) | −0.0007(13) |
| C1B | 0.0118(17) | 0.0161(18) | 0.0118(17) | −0.0027(14) | 0.0000(13) | −0.0026(14) |
| C2B | 0.0155(17) | 0.0143(17) | 0.0105(17) | −0.0022(14) | 0.0023(14) | −0.0002(14) |
| C3B | 0.0108(16) | 0.021(2) | 0.0184(19) | 0.0005(16) | −0.0009(14) | 0.0008(15) |
| C4B | 0.0182(18) | 0.0217(19) | 0.0101(17) | −0.0003(15) | −0.0002(14) | −0.0004(16) |
| C5B | 0.0219(19) | 0.0193(19) | 0.0098(17) | −0.0038(15) | 0.0015(15) | −0.0024(16) |
| C6B | 0.0107(16) | 0.023(2) | 0.0198(19) | −0.0040(17) | 0.0005(14) | −0.0008(15) |
| C7B | 0.0153(17) | 0.021(2) | 0.0140(18) | −0.0011(15) | −0.0018(14) | 0.0001(15) |
| C8B | 0.0168(18) | 0.0205(19) | 0.0153(18) | 0.0025(15) | 0.0034(15) | −0.0012(15) |
| C9B | 0.030(2) | 0.020(2) | 0.0137(19) | 0.0037(16) | 0.0000(16) | −0.0012(17) |
| C10B | 0.0230(19) | 0.0192(19) | 0.0157(18) | 0.0023(16) | −0.0045(15) | 0.0027(16) |
| C11B | 0.0153(18) | 0.0179(19) | 0.0189(19) | 0.0018(15) | −0.0041(15) | 0.0034(15) |
| C12B | 0.0146(18) | 0.039(3) | 0.020(2) | 0.0000(19) | 0.0067(15) | −0.0044(18) |
| Zn1C | 0.0128(2) | 0.0178(2) | 0.0167(2) | 0.00044(18) | 0.00144(16) | 0.00073(17) |
| Cl1C | 0.0193(4) | 0.0226(5) | 0.0174(4) | −0.0003(4) | 0.0029(4) | 0.0003(4) |
| Cl2C | 0.0193(4) | 0.0212(5) | 0.0244(5) | 0.0005(4) | −0.0017(4) | −0.0042(4) |
| Cl3C | 0.0222(4) | 0.0211(5) | 0.0180(4) | 0.0022(4) | 0.0017(4) | 0.0010(4) |
| N1C | 0.0196(18) | 0.031(2) | 0.0189(18) | −0.0016(15) | −0.0022(14) | 0.0049(15) |
| C1C | 0.021(2) | 0.020(2) | 0.021(2) | −0.0002(16) | 0.0020(17) | 0.0044(16) |
| C2C | 0.0155(19) | 0.027(2) | 0.022(2) | 0.0000(17) | −0.0005(16) | 0.0029(16) |

**Geometric parameters (Å, ^o^)**

| Zn1–Cl2 | 2.2202(10) | N3B–C11B | 1.479(5) |
| --- | --- | --- | --- |
| Zn1–O1A | 2.002(3) | C2B–C3B | 1.395(5) |
| Zn1–N1A | 2.207(3) | C2B–C7B | 1.400(5) |
| Zn1–O1B | 2.012(3) | C3B–H3B | 0.9500 |
| Zn1–N1B | 2.211(3) | C3B–C4B | 1.383(5) |
| O1A–C1A | 1.256(4) | C4B–H4B | 0.9500 |
| O2A–C5A | 1.354(4) | C4B–C5B | 1.392(5) |
| O2A–C12A | 1.434(5) | C5B–C6B | 1.399(5) |
| N1A–N2A | 1.267(4) | C6B–H6B | 0.9500 |
| N1A–C2A | 1.405(5) | C6B–C7B | 1.382(5) |
| N2A–C1A | 1.435(5) | C7B–H7B | 0.9500 |
| N3A–C1A | 1.309(5) | C8B–H8BA | 0.9900 |
| N3A–C8A | 1.484(5) | C8B–H8BB | 0.9900 |
| N3A–C11A | 1.493(4) | C8B–C9B | 1.535(5) |
| C2A–C3A | 1.399(5) | C9B–H9BA | 0.9900 |
| C2A–C7A | 1.406(5) | C9B–H9BB | 0.9900 |
| C3A–H3A | 0.9500 | C9B–C10B | 1.527(6) |
| C3A–C4A | 1.383(5) | C10B–H10C | 0.9900 |
| C4A–H4A | 0.9500 | C10B–H10D | 0.9900 |
| C4A–C5A | 1.392(5) | C10B–C11B | 1.521(5) |
| C5A–C6A | 1.412(5) | C11B–H11C | 0.9900 |
| C6A–H6A | 0.9500 | C11B–H11D | 0.9900 |
| C6A–C7A | 1.367(6) | C12B–H12D | 0.9800 |
| C7A–H7A | 0.9500 | C12B–H12E | 0.9800 |
| C8A–H8AA | 0.9900 | C12B–H12F | 0.9800 |
| C8A–H8AB | 0.9900 | Zn1C–Cl1C | 2.2408(10) |
| C8A–C9A | 1.529(5) | Zn1C–Cl2C | 2.2368(11) |
| C9A–H9AA | 0.9900 | Zn1C–Cl3C | 2.2435(10) |
| C9A–H9AB | 0.9900 | Zn1C–N1C | 2.095(3) |
| C9A–C10A | 1.536(5) | N1C–C1C | 1.149(5) |
| C10A–H10A | 0.9900 | C1C–C2C | 1.438(5) |
| C10A–H10B | 0.9900 | C2C–H2CA | 0.9800 |
| C10A–C11A | 1.527(5) | C2C–H2CB | 0.9800 |
| C11A–H11A | 0.9900 | C2C–H2CC | 0.9800 |
| C11A–H11B | 0.9900 | N1B–N2B | 1.270(4) |
| C12A–H12A | 0.9800 | N1B–C2B | 1.407(5) |
| C12A–H12B | 0.9800 | N2B–C1B | 1.439(5) |
| C12A–H12C | 0.9800 | N3B–C1B | 1.311(5) |
| O1B–C1B | 1.262(4) | O2B–C12B | 1.440(5) |
| O2B–C5B | 1.356(5) | N3B–C8B | 1.484(5) |
| O1A–Zn1–Cl2 | 126.20(9) | C8A–C9A–H9AA | 111.0 |
| O1A–Zn1–N1A | 75.27(11) | C8A–C9A–H9AB | 111.0 |
| O1A–Zn1–O1B | 118.48(11) | C8A–C9A–C10A | 103.9(3) |
| O1A–Zn1–N1B | 93.31(11) | H9AA–C9A–H9AB | 109.0 |
| N1A–Zn1–Cl2 | 103.62(9) | C10A–C9A–H9AA | 111.0 |
| N1A–Zn1–N1B | 160.92(12) | C10A–C9A–H9AB | 111.0 |
| O1B–Zn1–Cl2 | 115.10(8) | C9A–C10A–H10A | 111.0 |
| O1B–Zn1–N1A | 96.41(11) | C9A–C10A–H10B | 111.0 |
| O1B–Zn1–N1B | 75.36(11) | H10A–C10A–H10B | 109.0 |
| N1B–Zn1–Cl2 | 95.47(8) | C11A–C10A–C9A | 104.0(3) |
| C1A–O1A–Zn1 | 115.6(2) | C11A–C10A–H10A | 111.0 |
| C5A–O2A–C12A | 117.8(3) | C11A–C10A–H10B | 111.0 |
| N2A–N1A–Zn1 | 115.1(2) | N3A–C11A–C10A | 103.0(3) |
| N2A–N1A–C2A | 116.2(3) | N3A–C11A–H11A | 111.2 |
| C2A–N1A–Zn1 | 128.6(2) | N3A–C11A–H11B | 111.2 |
| N1A–N2A–C1A | 110.5(3) | C10A–C11A–H11A | 111.2 |
| C1A–N3A–C8A | 120.6(3) | C10A–C11A–H11B | 111.2 |
| C1A–N3A–C11A | 127.2(3) | H11A–C11A–H11B | 109.1 |
| C8A–N3A–C11A | 112.1(3) | O2A–C12A–H12A | 109.5 |
| O1A–C1A–N2A | 123.5(3) | O2A–C12A–H12B | 109.5 |
| O1A–C1A–N3A | 123.0(3) | O2A–C12A–H12C | 109.5 |
| N3A–C1A–N2A | 113.5(3) | H12A–C12A–H12B | 109.5 |
| N1A–C2A–C7A | 122.3(3) | H12A–C12A–H12C | 109.5 |
| C3A–C2A–N1A | 117.3(3) | H12B–C12A–H12C | 109.5 |
| C3A–C2A–C7A | 120.5(4) | C1B–O1B–Zn1 | 113.8(2) |
| C2A–C3A–H3A | 119.9 | C5B–O2B–C12B | 117.5(3) |
| C4A–C3A–C2A | 120.2(3) | N2B–N1B–Zn1 | 113.7(2) |
| C4A–C3A–H3A | 119.9 | N2B–N1B–C2B | 116.1(3) |
| C3A–C4A–H4A | 120.3 | C2B–N1B–Zn1 | 128.0(2) |
| C3A–C4A–C5A | 119.4(4) | N1B–N2B–C1B | 110.2(3) |
| C5A–C4A–H4A | 120.3 | C1B–N3B–C8B | 122.1(3) |
| O2A–C5A–C4A | 125.4(4) | C1B–N3B–C11B | 126.3(3) |
| O2A–C5A–C6A | 114.4(3) | C11B–N3B–C8B | 111.7(3) |
| C4A–C5A–C6A | 120.2(4) | O1B–C1B–N2B | 123.9(3) |
| C5A–C6A–H6A | 119.7 | O1B–C1B–N3B | 123.0(3) |
| C7A–C6A–C5A | 120.6(3) | N3B–C1B–N2B | 113.1(3) |
| C7A–C6A–H6A | 119.7 | C3B–C2B–N1B | 117.2(3) |
| C2A–C7A–H7A | 120.4 | C3B–C2B–C7B | 120.3(3) |
| C6A–C7A–C2A | 119.2(4) | C7B–C2B–N1B | 122.5(3) |
| C6A–C7A–H7A | 120.4 | C2B–C3B–H3B | 120.2 |
| N3A–C8A–H8AA | 111.2 | C4B–C3B–C2B | 119.6(3) |
| N3A–C8A–H8AB | 111.2 | C4B–C3B–H3B | 120.2 |
| N3A–C8A–C9A | 103.0(3) | C3B–C4B–H4B | 120.1 |
| H8AA–C8A–H8AB | 109.1 | C3B–C4B–C5B | 119.8(4) |
| C9A–C8A–H8AA | 111.2 | C5B–C4B–H4B | 120.1 |
| C9A–C8A–H8AB | 111.2 | O2B–C5B–C4B | 115.6(4) |
| N3B–C11B–C10B | 102.6(3) | O2B–C5B–C6B | 123.5(3) |
| N3B–C11B–H11C | 111.3 | C4B–C5B–C6B | 120.9(4) |
| N3B–C11B–H11D | 111.3 | C5B–C6B–H6B | 120.5 |
| C10B–C11B–H11C | 111.3 | C7B–C6B–C5B | 119.0(3) |
| C10B–C11B–H11D | 111.3 | C7B–C6B–H6B | 120.5 |
| H11C–C11B–H11D | 109.2 | C2B–C7B–H7B | 119.9 |
| O2B–C12B–H12D | 109.5 | C6B–C7B–C2B | 120.2(4) |
| O2B–C12B–H12E | 109.5 | C6B–C7B–H7B | 119.9 |
| O2B–C12B–H12F | 109.5 | N3B–C8B–H8BA | 111.2 |
| H12D–C12B–H12E | 109.5 | N3B–C8B–H8BB | 111.2 |
| H12D–C12B–H12F | 109.5 | N3B–C8B–C9B | 102.9(3) |
| H12E–C12B–H12F | 109.5 | H8BA–C8B–H8BB | 109.1 |
| Cl1C–Zn1C–Cl3C | 119.15(4) | C9B–C8B–H8BA | 111.2 |
| Cl2C–Zn1C–Cl1C | 112.42(4) | C9B–C8B–H8BB | 111.2 |
| Cl2C–Zn1C–Cl3C | 113.01(4) | C8B–C9B–H9BA | 111.0 |
| N1C–Zn1C–Cl1C | 101.12(10) | C8B–C9B–H9BB | 111.0 |
| N1C–Zn1C–Cl2C | 108.91(11) | H9BA–C9B–H9BB | 109.0 |
| N1C–Zn1C–Cl3C | 100.19(10) | C10B–C9B–C8B | 103.9(3) |
| C1C–N1C–Zn1C | 164.7(4) | C10B–C9B–H9BA | 111.0 |
| N1C–C1C–C2C | 178.4(5) | C10B–C9B–H9BB | 111.0 |
| C1C–C2C–H2CA | 109.5 | C9B–C10B–H10C | 111.2 |
| C1C–C2C–H2CB | 109.5 | C9B–C10B–H10D | 111.2 |
| C1C–C2C–H2CC | 109.5 | H10C–C10B–H10D | 109.2 |
| H2CA–C2C–H2CB | 109.5 | C11B–C10B–C9B | 102.6(3) |
| H2CA–C2C–H2CC | 109.5 | C11B–C10B–H10C | 111.2 |
| H2CB–C2C–H2CC | 109.5 | C11B–C10B–H10D | 111.2 |

**2.NMR spectra**


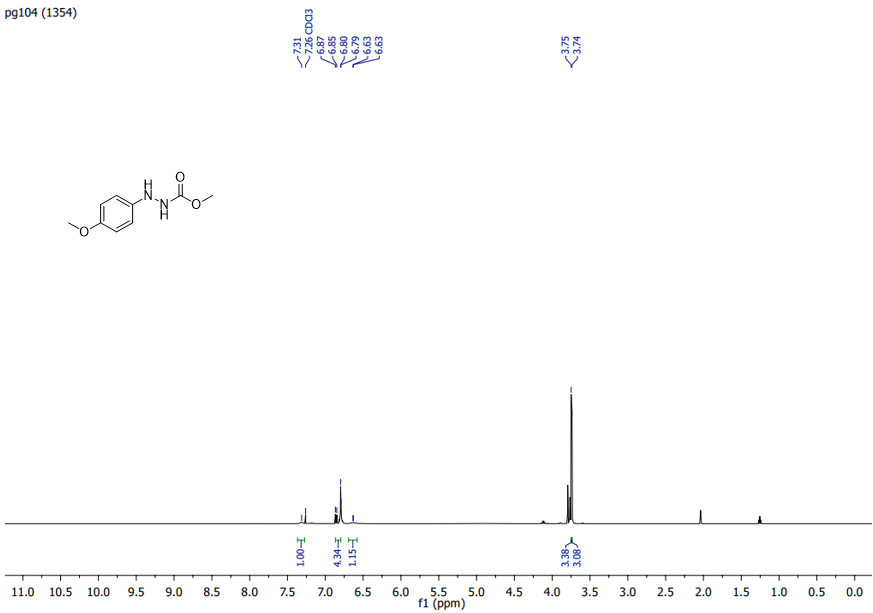


**Fig S1:** ^1^H NMR of **(3)** Methyl 2-(4-methoxyphenyl)hydrazine-1-carboxylate in CDCl_3_


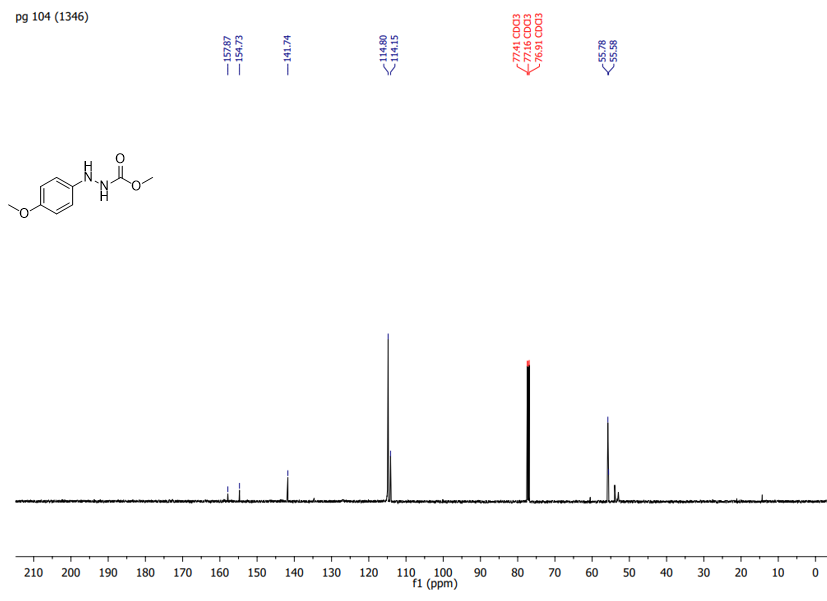


**Fig S2:** ^13^C NMR of **(3)** Methyl 2-(4-methoxyphenyl)hydrazine-1-carboxylate in CDCl_3_


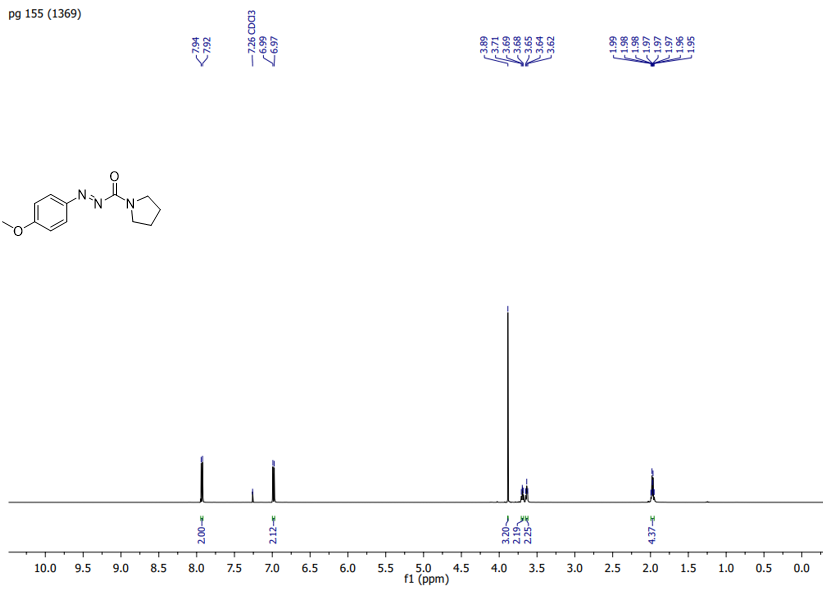


**Fig S3:** ^1^H NMR of (**4)** Pyrrolidnyl-4-methoxyphenylazoformamide in CDCl_3_


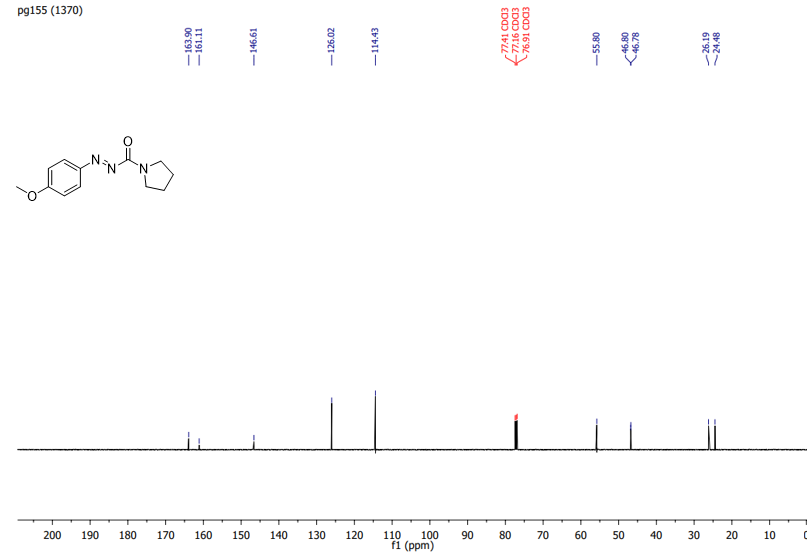


**Fig S4:** ^13^C NMR of (**4)** Pyrrolidnyl-4-methoxyphenylazoformamide in CDCl_3_

_
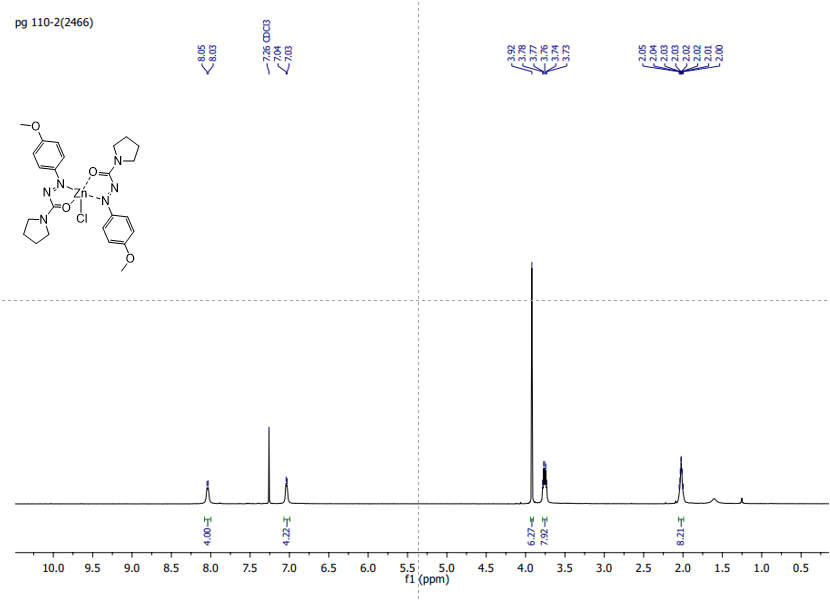
_

**Fig S5:** ^1^H NMR of (**1)** title Zn complex in CDCl_3_

**
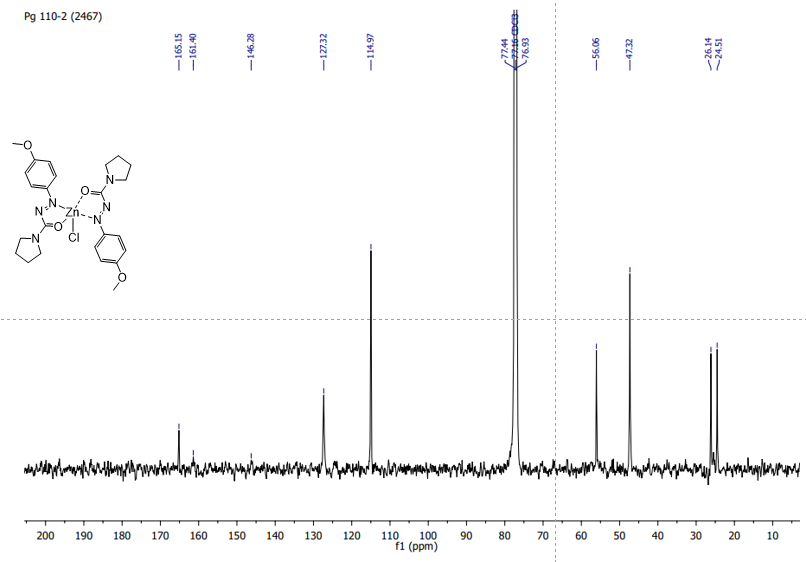
**

**Fig S6:** ^13^C NMR of (**1)** title Zn complex in CDCl_3_
